# Rock Inhibitor Y-27632 Enables Feeder-Free, Unlimited Expansion of *Sus scrofa domesticus* Swine Airway Stem Cells to Facilitate Respiratory Research

**DOI:** 10.1155/2019/3010656

**Published:** 2019-11-23

**Authors:** Tina P. Dale, Emily Borg D'anastasi, Mohammed Haris, Nicholas R. Forsyth

**Affiliations:** ^1^School of Pharmacy and Bioengineering, Faculty of Medicine and Health Sciences, Keele University, Guy Hilton Research Centre, Thornburrow Drive, Stoke-on-Trent, Staffordshire ST4 7QB, UK; ^2^University Hospitals of North Midlands NHS Trust, Royal Stoke University Hospital, Newcastle Road, Stoke-on-Trent, Staffordshire ST4 6QG, UK

## Abstract

Current limitations in the efficacy of treatments for chronic respiratory disorders position them as prospective regenerative medicine therapeutic targets. A substantial barrier to these ambitions is that research requires large numbers of cells whose acquisition is hindered by the limited availability of human tissue samples leading to an overreliance on physiologically dissimilar rodents. The development of cell culture strategies for airway cells from large mammals will more effectively support the transition from basic research to clinical therapy. Using readily available porcine lungs, we isolated conducting airway tissue and subsequently a large number of porcine airway epithelial cells (pAECs) using a digestion and mechanical scraping technique. Cells were cultured in a variety of culture media formulations, both foetal bovine serum-containing and serum-free media, in air (21%) and physiological (2%) oxygen tension and in the presence and absence of Rho kinase inhibitor Y-27362 (RI). Cell number at isolation and subsequent population doublings were determined; cells were characterised during culture and following differentiation by immunofluorescence, histology, and IL-8 ELISA. Cells were positive for epithelial markers (pan-cytokeratin and E-cadherin) and negative for fibroblastic markers (vimentin and smooth muscle actin). Supplementation of cultures with Y-27632 allowed for unlimited expansion whilst sustaining an epithelial phenotype. Early passage pAECs readily produced differentiated air-liquid interface (ALI) cultures with a capacity for mucociliary differentiation retained after substantial expansion, strongly modulated by the culture condition applied. Primary pAECs will be a useful tool to further respiratory-oriented research whilst RI-expanded pAECs are a promising tool, particularly with further optimisation of culture conditions.

## 1. Introduction

The conducting airways are lined with a pseudostratified epithelial layer consisting predominantly of ciliated and secretory cells. These are responsible for airway functionality and are supported by underlying basal cells which are responsible for the homeostasis and regeneration of the airways [1]. A plentiful source of primary airway epithelial cells (AECs) is critical for the study of airway dysfunction during disease [2–4], to support the development of representative airway models for drug screening, i.e., inhaled chemotherapeutics [5], and as a key component in the development of regenerative medicine approaches including cell therapy and tissue engineering [6].

To date, the majority of research in the field has been carried out with readily available cell lines with a malignant origin or with rodent primary cells which display differences in the distribution and identity of cell populations when compared to those found in human airways [1]. Human primary cells from large and small airways are now commercially available; however, these come at high cost, in limited quantities and from a limited pool of donors. Alternatively, there are genetically modified, immortalised cell lines such as NL20 (ATCC CRL-2503). These have the advantage of essentially unlimited expansion capacity but also represent only a single individual and do not recapitulate normal biology. The development of cell lines from alternative mammalian sources would therefore be advantageous. Porcine lungs and their associated cells have a number of desirable characteristics. Their availability and low cost as a by-product of the meat-producing industry supports the use of multiple donor animals, whilst still reducing the number of animals sacrificed for research purposes only. Additionally, the size of the lungs would support research of increasing complexity, with multiple cell types, from a single donor animal. Although evolutionarily distinct from primates, pig lung physiology more closely mimics that of the human [7–10]. Taken together, this means that the development of porcine cell lines would facilitate the translation of research from the laboratory setting to large animal models and clinical therapies more effectively, with further support from the ongoing development of humanised pig tissues [11]. A number of tools supporting these developments have emerged including the publication of the pig genome and development of targeted genetic modification in these animals allowing the development of cystic fibrosis animal models [12].

The successful culture of airway epithelial cells under normal culture conditions is reliant on the presence initially of a sufficient number of airway stem cells and their subsequent proliferation. The basal cells of the airway are a stem or progenitor cell type, differentiating under appropriate conditions into multiple airway cell types that form the pseudostratified epithelium that lines the airway, including ciliated and secretory (predominantly goblet) cells, and which under normal conditions are responsible for the maintenance and regeneration of the airway epithelium in vivo [1]. Whilst it is possible to culture-expand basal cells to an extent, they rapidly enter replicative senescence under standard culture conditions. A number of strategies have been applied in order to extend cellular replicative capacity including gene transfer with SV40 T-antigen [13], HPV-16 E6 and E7 [14], and the catalytic subunit of telomerase, TERT [15].

An alternative technique that does not involve direct genetic manipulation of the cells is the application of Rho-associated coiled coil protein kinase (ROCK) inhibitor (RI), in combination with inactivated fibroblast feeders. ROCK inhibition, generally with Y-27632, an inhibitor of ROCK1 and ROCK2, was initially exploited for its positive effects on the survival of dissociated embryonic stem cells [16] and has since been used to enhance the proliferation of a number of epithelial cell types including keratinocytes [17], prostate and breast cells [18], and nonepithelial cell types from the intervertebral disc [19] in a process generally referred to as conditional immortalisation or conditional reprogramming, producing conditionally reprogrammed cells (CRCs). Rho-associated protein kinases have roles in numerous cellular functions including cell migration and adhesion, proliferation, differentiation, and apoptosis [20].

Herein, we have used a readily available source of cells from the domestic pig (*Sus scrofa domesticus*), a more biologically relevant model than those derived from rodents, in combination with Rho-associated kinase (ROCK) inhibitor Y-27632 that enables unlimited, feeder-free expansion of porcine airway epithelial cells.

## 2. Materials and Methods

### 2.1. Primary Airway Cell Isolation and Culture

Whole porcine lungs were obtained directly from an abattoir within hours of animal death. Tracheal tissue was first removed and discarded. Conducting airways were then dissected free of the surrounding lung parenchymal tissue, cut into short sections, and washed in three changes of 500 *μ*g/mL gentamycin, 100 *μ*g/mL ciprofloxacin, 1000 IU/mL penicillin, 1 mg/mL streptomycin, and 2.5 *μ*g/mL amphotericin B in Hank's balanced salt solution (HBSS). Segments of airway tissue were digested overnight on a rocker at 4°C in 1 mg/mL protease XIV, 0.005% trypsin, and 10 ng/mL DNAse I in F12 : DMEM (1 : 1). Airway sections were then cut open along the lumen, and the cells lining the lumenal surface of the airways were scraped off with a sterile scalpel and transferred to HBSS. Cells were centrifuged at 350 g for 3 minutes and seeded on tissue culture plastic that was either uncoated or precoated with type 1 collagen (10 *μ*g/cm^2^ type 1 rat tail collagen solution (Corning)). Cells were then incubated in either standard tissue culture incubators (21% O_2_ (air), 5% CO_2_, 5% humidity, 37°C) or tri-gas incubators (2% O_2_, 5% CO_2_, 73% N_2_, 5% humidity, 37°C) as indicated.

Tissue-isolated cells were plated in four differing media formulations: three FBS-containing media: (1) cFAD [21]: DMEM : F12 (75 : 25), 10% (*v*/*v*) FBS, 2 mM L-glutamine, 24 *μ*g/mL adenine, 5 *μ*g/mL transferrin, 5 *μ*g/mL insulin, 0.4 *μ*g/mL hydrocortisone, 0.13 *μ*g/mL triiodothyronine, and 10 ng/mL epidermal growth factor (EGF); (2) reduced cFAD (rcFAD): Ham's F12, 4% (*v*/*v*) FBS, 1 mM L-glutamine, 1 *μ*g/mL transferrin, 5 *μ*g/mL insulin, 0.5 *μ*g/mL hydrocortisone, and 10 ng/mL epidermal growth factor (EGF); (3) DMEM: 10% (*v*/*v*) FBS and 2 mM L-glutamine; and (4) one commercial, epithelial, serum-free medium: LHC-9 (Gibco). An additional commercial, serum-free, epithelial medium, PromoCell Airway Epithelial Cell Growth Medium (LA), was also utilised in support of long-term expansion. All media were supplemented with 100 IU/mL penicillin, 100 *μ*g/mL streptomycin, 0.25 *μ*g/mL amphotericin B, 50 *μ*g/mL gentamycin, 10 *μ*g/mL ciprofloxacin, and 1% (*v*/*v*) nonessential amino acids (NEAA), where indicated media were also supplemented with Y-27632 RI at 10 *μ*M.

Media changes were performed twice weekly and when necessary cells were subcultured enzymatically at preconfluence using 0.05% (*w*/*v*) trypsin/0.02% (*w*/*v*) ethylenediaminetetraacetic acid (EDTA) (5 g/L trypsin and 2 g/L EDTA diluted 1 in 10 in calcium- and magnesium-free PBS).

### 2.2. Cell Line Culture

Cell lines (A549 and BEAS-2B) were maintained in DMEM supplemented with 10% (*v*/*v*) FBS, 1% (*v*/*v*) NEAA, 2 mM L-glutamine, 100 IU/mL penicillin, 100 *μ*g/mL streptomycin, and 0.25 *μ*g/mL amphotericin B. Media changes were performed twice weekly and when necessary cells were subcultured enzymatically at preconfluence as per primary cells.

### 2.3. Immunocytochemistry

For immunofluorescence, cells were fixed in either 95% (*w*/*v*) chilled methanol or 10% (*w*/*v*) neutral-buffered formalin and stored under PBS at 4°C until staining. Cells were permeabilised with 0.1% (*w*/*v*) Triton X for 10 minutes. Fixed cells were incubated with 1% (*w*/*v*) bovine serum albumin for 1 hour at room temperature before overnight incubation with primary antibody diluted in PBS at 4°C (vimentin, 1 in 500 (ab92547); pan-cytokeratin, 1 in 100 (ab86734); E-cadherin, 1 in 50 (ab15148); P63, 1 in 300 (ab124762); alpha smooth muscle actin, 1 in 100 (ab5694); and beta IV tubulin, 1 in 500 (ab179509)). Antibody was then removed, and cells were washed in 3 changes of PBS prior to incubation with secondary antibody (DyLight 488 anti-rabbit IgG (ab96881) or DyLight 594 anti-mouse IgG (ab96881)) for 1 hour at room temperature in the dark. Secondary was then removed, and cells were washed in 2 changes of PBS before incubation with DAPI for 5 minutes. Cells were then washed once and imaged on a Nikon Eclipse T1 fluorescence microscope with a Nikon DSi 1 camera.

### 2.4. Air-Liquid Interface Cultures

To induce differentiation, cells were cultured at air-liquid interface on ThinCert cell culture inserts (0.34 *μ*m area, 0.45 *μ*m pore size) (Greiner). Cells were seeded at 2 × 10^5^ cells/cm^2^ in cFAD media, with or without RI as indicated. Cultures were maintained for 2-4 days until monolayers were confluent; then, culture medium was removed and replaced with complete PneumaCult ALI (Stemcell Technologies) medium in the basal compartment only. Medium was then changed three times per week for the duration of culture.

### 2.5. Transepithelial Electrical Resistance Measurements

Transepithelial electrical resistance was determined for ALI cultures using a Millicell ERS2 epithelial volt-ohm meter. To measure TEER, medium was removed from culture inserts and wells and replaced with HBSS with calcium and magnesium (1.25 mL basal compartment, 250 *μ*L apical compartment), and resistance was determined by inserting the probe across the apical and basal compartments. HBSS was then aspirated, and media returned to the basal compartments of cultures only, to restore ALI. Resistance was calculated by subtracting the resistance measurement of HBSS and insert only from ALI measurements and multiplying by the insert culture area to give an *Ω*·cm^2^ value.

### 2.6. Organoid Culture

Organoids were prepared in 96-well sterile suspension culture plates using a modification of the Stemcell Technologies protocol associated with the PneumaCult ALI medium. Wells were coated with 35 *μ*L of 40% (*v*/*v*) Matrigel in PneumaCult medium at 4°C. This was transferred to a 37°C incubator for approximately 30 minutes to set. Cells were removed from culture plates using trypsin/EDTA and resuspended as a single-cell suspension in 5% (*v*/*v*) Matrigel in complete PneumaCult ALI medium. 90 *μ*L of suspension (17,000 cells) overlaid on the preset 40% Matrigel and the plate returned to the incubator. Medium was changed 3 times per week by removing 75 *μ*L and replacing with 100 *μ*L of complete PneumaCult ALI.

Organoids were harvested by gentle pipetting and washed in 2 changes of phosphate-buffered saline prior to fixation in 4% (*w*/*v*) paraformaldehyde for 30 minutes. Formalin was removed, and organoids were suspended in prewarmed HistoGel which was then allowed to cool and set at room temperature. HistoGel blocks were serially dehydrated in graded alcohol (70% (*v*/*v*) industrial methylated spirits (IMS), 90% (*v*/*v*) isopropanol, 100% (*v*/*v*) isopropanol, and 100% (*v*/*v*) isopropanol) prior to wax embedding and sectioning at 7 *μ*m.

### 2.7. Alcian Blue-Periodic Acid Schiff (PAS) Staining

PBS was removed from basal and apical chambers of fixed ALI cultures. Sections of lung organoids were dewaxed with 2 changes of 100% xylene, followed by rehydration in graded alcohol and a final wash with distilled water. Samples were covered with 1% Alcian blue in 3% aqueous acetic acid for 30 minutes, washed with distilled water until water remained clear followed by 0.5% periodic acid solution for 5 minutes, washed with distilled water, and covered with Schiff reagent for 20 minutes. Schiff reagent was removed, and the samples were washed with lukewarm tap water for 5 minutes. Culture inserts were imaged submerged with distilled water; organoid sections were dehydrated and mounted in DPX.

### 2.8. LPS Stimulation of Cells

Cells at passage 30 cultured in cFAD+RI were plated in collagen coated (type I rat tail, 10 *μ*g/cm^2^) 48-well tissue culture plates at 75,000 cells/cm^2^ and allowed to attach overnight. Medium was then removed and replaced with 350 *μ*L of medium (DMEM (4.5 g/L glucose), 5% (*v*/*v*) FBS, 1% (*v*/*v*) L-Glut, 1% NEAA) supplemented with LPS at concentrations of 0 to 100 *μ*g/mL. After 24 hours, medium was harvested, centrifuged at 600 g to remove cell debris, and stored at -80°C.

### 2.9. Interleukin-8 ELISA

IL-8 production in response to LPS stimulation was quantified using a Swine IL-8 ELISA kit (Invitrogen) according to manufacturer's instructions. All reagents were brought to room temperature prior to carrying out the assay; all washes were performed 4 times with wash buffer. 50 *μ*L of incubation buffer was added to all wells; sample was added to each well and incubated for 2 hours at room temperature; well contents were then aspirated; and wells were washed. Biotinylated swine IL-8 was added to all wells and incubated for 1 hour at room temperature prior to aspiration and washing. Streptavidin-HRP was added to all wells, incubated for 30 minutes at room temperature, aspirated, and washed. Stabilised chromogen was then added to all wells and incubated for 30 minutes in the dark at room temperature, after which stop solution was added to all wells and absorbance was read at 450 nm. Sample IL-8 concentrations were determined by linear regression from a swine IL-8 standard curve.

### 2.10. Statistical Analysis

Data are expressed throughout as mean ± standard deviation; all statistical analysis was carried out using GraphPad Prism 6.01 (GraphPad, San Diego, California, USA). Results were deemed to be statistically significant when *p* ≤ 0.05. The number of cells recovered was compared by two-way ANOVA with Tukey's or Sidak's multiple comparison pairwise analysis. TEER in RI- versus non-RI-supplemented cultures was compared by two-way ANOVA with Sidak's multiple comparison pairwise analysis. IL-8 ELISA results were compared using two-way ANOVA of nonnormalised data and with pairwise analysis of 2% and 21% O_2_ by Sidak's multiple comparison test. ELISA data normalised to LPS-untreated IL-8 values were subject to two-way ANOVA and compared using Dunnett's multiple comparison test of LPS-treated versus LPS-untreated cells.

## 3. Results

**Figure 1:**
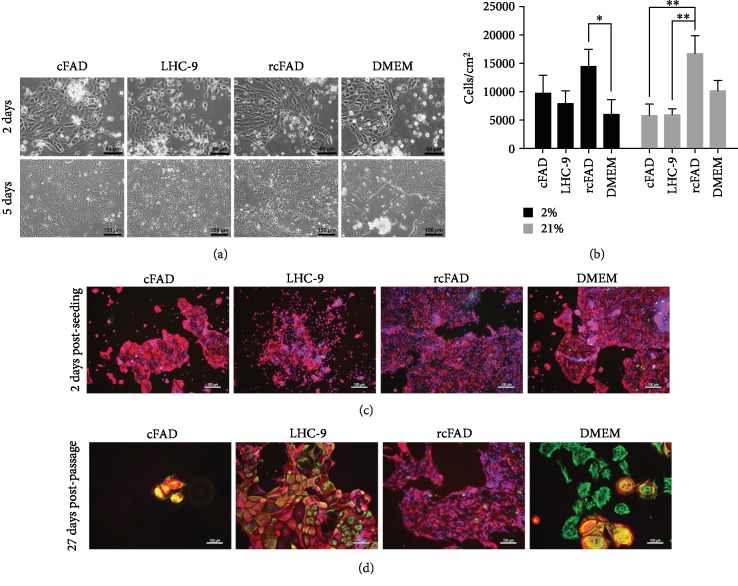
pAEC recovery in variable media and oxygen conditions. Culture of pAECs in differing oxygen levels has no effect on cell numbers during recovery; proliferative epithelial colonies with similar morphology were recovered in all media conditions. (a) Phase contrast images of cells at 2 days (upper) and 5 days (lower) postseeding in the 4 media types; images are of 21% O_2_ cells and are representative of both O_2_ levels. Scale bar 80 *μ*m upper, 150 *μ*m lower. (b) Cells per cm^2^ after 5 days of culture in all media types and both O_2_ conditions. Data are mean ± standard deviation, *n* = 3. ^∗^*p* ≤ 0.05 and ^∗∗^*p* ≤ 0.01. (c) Immunofluorescent images of recovered pAECs stained with pan-cytokeratin (red) and vimentin (green) 2 days after seeding, DAPI (blue) nuclear counterstain; images are of 21% O_2_ cells and are representative of both O_2_ levels. Scale bar 100 *μ*m. (d) Immunofluorescent images of cultured cells stained with pan-cytokeratin (red) and vimentin (green) 27 days after passaging recovered cells, DAPI (blue) nuclear counterstain; images are of 21% O_2_ cells and are representative of both O_2_ levels. Scale bar 100 *μ*m.

To establish culture of pAECs, primary cells isolated from porcine airways were plated in 4 different media formulations (cFAD, LHC-9, rcFAD, and DMEM). All 4 formulations supported the isolation of cells with a typical epithelial cuboidal morphology at day 2 in both 2% and 21% oxygen (Figure 1(a)), top). cFAD, rcFAD, and DMEM cells were primarily in multicell colonies, whilst the commercial media LHC-9 predominantly supported isolation single cells. Cell counts at day 5 (Figure 1(b)) indicated that in both 2% and 21% O_2_, rcFAD recovered the largest number of cells (14433 ± 3028 cells/cm^2^ and 16720 ± 3178 cells/cm^2^, respectively), achieving significance over DMEM in 2% O_2_ (*p* ≤ 0.05) and both cFAD and LHC-9 in 21% O_2_ (*p* ≤ 0.01). Within each media type, no significant difference was seen in cell numbers between 2% and 21% O_2_ culture. By day 5, differences in cell morphology across the 4 media types had become apparent (Figure 1(a)), bottom) with retention of the cobblestone in LHC-9 and rcFAD and a flattened appearance in DMEM, whilst cFAD cell morphology was variable. Cells were fixed and immunostained 2 days after seeding with pan-cytokeratin and vimentin (Figure 1(c)) to determine the proportions of epithelial versus fibroblastic cells recovered. At this early stage, cells were stained almost exclusively with pan-cytokeratin, indicating a relatively pure population of epithelial cells in all media types. Postpassage cells recovered poorly, failing to reach confluence in any condition with a large increase in vimentin staining in all but rcFAD (Figure 1(d)).

**Figure 2:**
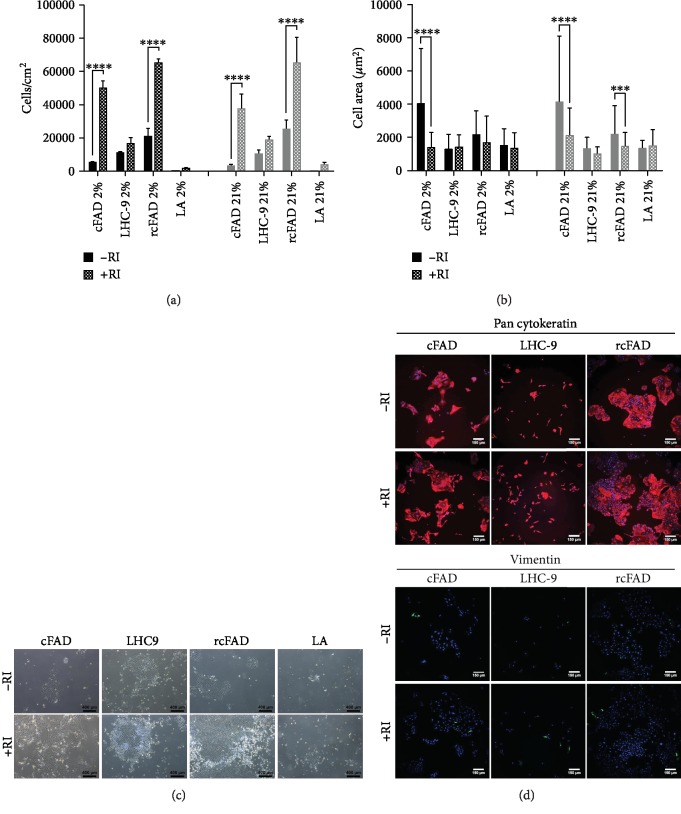
RI supplementation significantly improves cell numbers and reduces cell size in FBS-containing media. (a) Cells per cm^2^ after culture in all media types and both O_2_ conditions with and without RI supplementation. Data are mean ± standard deviation, *n* = 3. ^∗∗∗∗^*p* ≤ 0.0001. (b) Cell area (*μ*m^2^) of cells following culture in all media types and both O_2_ conditions with and without RI supplementation. Data are mean ± standard deviation, *n* = 3. ^∗∗∗^*p* ≤ 0.001 and ^∗∗∗∗^*p* ≤ 0.0001. (c) Phase contrast images of cells postseeding in the 4 media types with and without RI supplementation; images are of 21% O_2_ cells and are representative of both O_2_ levels. Scale bar 400 *μ*m. (d) Immunofluorescent images of primary cell cultures in the indicated media conditions stained with pan-cytokeratin (red, upper panel) and vimentin (green, lower panel), both with DAPI (blue) nuclear counterstaining; images are of 21% O_2_ cells and are representative of both O_2_ levels. Scale bar 150 *μ*m.

Cell culture media were subsequently supplemented with 10 *μ*M Y-27632 along with the addition of a type I collagen culture vessel coating to explore potential improvements in cell yield and expansion potential. As epithelial cells cannot be cultured long term in DMEM supplemented with 10% FBS without undergoing squamous terminal differentiation [22], this media formulation was replaced with a second commercial epithelial medium: Airway Epithelial Cell Growth Medium for large airways (LA, PromoCell). In all cases, the presence of RI increased early expanded cell numbers (Figure 2(a)), ranging from a 1.5-fold increase in LHC-9 2% O_2_ to a 17.2-fold increase in LA medium in 21% O_2_. Significant increases in cell numbers were noted in both cFAD and rcFAD, 2% and 21% O_2_ (*p* ≤ 0.0001), but not with either LHC-9 or LA media. In the absence of RI, cells were significantly larger in cFAD in both 2% (2.9-fold larger, *p* ≤ 0.0001) and 21% O_2_ (2.0-fold larger, *p* ≤ 0.0001) and rcFAD 21% O_2_ (1.5-fold, *p* ≤ 0.001) (Figures 2(b) and 2(c)). The LA media promoted a cell morphology and comparable size to LHC-9 recovered cells but in greatly reduced numbers. Pan-cytokeratin and vimentin expression levels indicated that cells were almost exclusively positive for pan-cytokeratin with no difference seen in RI-supplemented cells at recovery (Figure 2(d)).

**Figure 3:**
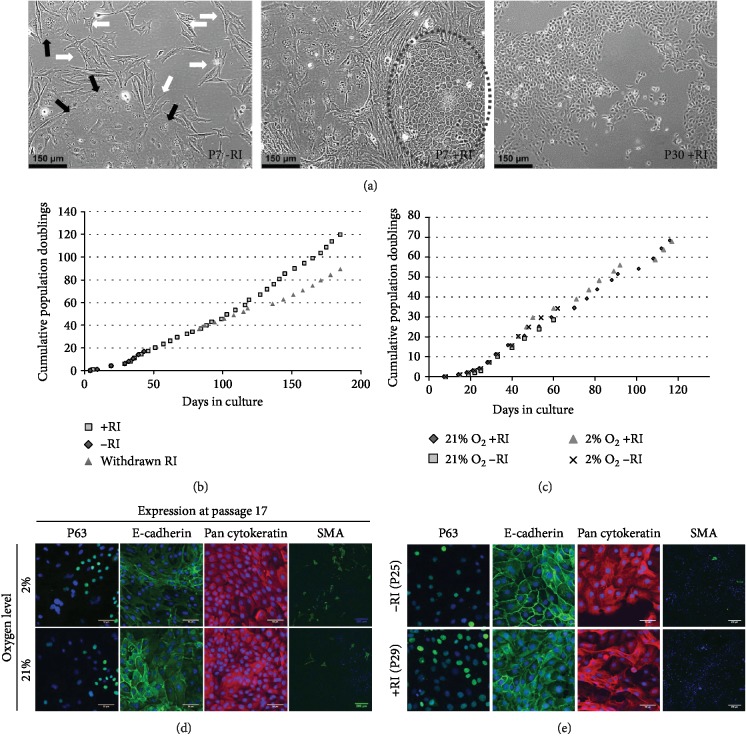
RI supplementation supported essentially unlimited, feeder-free proliferation of cells with a sustained epithelial morphology. (a) Phase contrast images of cells. Left: passage 7 cells cultured absent RI; white arrows indicate fibroblastic cells; black arrows indicate squamous cells. Middle: passage 7 cells cultured with RI; an epithelial colony is circled. Right: passage 30 cells cultured with RI continuously. Scale bar 150 *μ*m. (b) Estimated cumulative population doublings in cells cultured without RI (blue diamond), with RI continuously (red squares), and with RI withdrawn from culture at the indicated time point (green triangles). (c) Estimated cumulative population doublings in cultures in 2% and 21% O_2_, with and without RI. (d) Immunofluorescent images of cultured cells at passage 17 stained with P63, E-cadherin, pan-cytokeratin, and smooth muscle actin (SMA) following culture in 2% (upper) or 21% (lower) O_2_, DAPI (blue) nuclear counterstain. Scale bar: P63, E-cadherin, and pan-cytokeratin 50 *μ*m; SMA 200 *μ*m. (e) Immunofluorescent images of cultured cells stained with P63, E-cadherin, pan-cytokeratin, and smooth muscle actin (SMA) following withdrawal of RI for more than 10 passages (upper) or with continuous RI supplementation (lower), DAPI (blue) nuclear counterstain. Scale bar: P63, E-cadherin, and pan-cytokeratin 50 *μ*m; SMA 200 *μ*m.

Cells from each condition were then expanded following standard cell culture practice with and without continued RI supplementation. LHC-9 and LA cultured cells underwent limited and slow proliferation before adopting a senescent morphology. rcFAD cultures maintained proliferation and a cobblestone morphology but developed poor substrate attachment over continued passage. Culture expansion in cFAD in the absence of RI resulted in rapid loss of epithelial cell morphology with cells adopting either a fibroblastic or an enlarged squamous appearance (Figure 3(a)), left) which over time resulted in an entirely fibroblastic population. RI-supplemented cultures also contained fibroblastic and squamous cells alongside a persistent population of cells with a distinct epithelial, cobblestone morphology (Figure 3, middle panel, oval). This later population of cells had become uniformly present by passages 10-13 (Figure 3(a)), right). Strikingly, the withdrawal of RI supplementation from cells resulted in a slowing but not a cessation of proliferation (Figure 3(b)); little difference was observed in the overall rate of proliferation between the culture conditions (Figure 3(c)). Additionally, no difference was seen in staining (Figure 3(d)) with P63, E-cadherin, pan-cytokeratin, or SMA expression by immunostaining following culture in both 2% and 21% O_2_ for 17 passages. Interestingly, and in contrast to cells never exposed to RI, following the withdrawal of RI, in addition to sustained if slowed proliferation, cells maintained their epithelial morphology and continued to express P63, E-cadherin, and pan-cytokeratin (Figure 3(e)).

**Figure 4:**
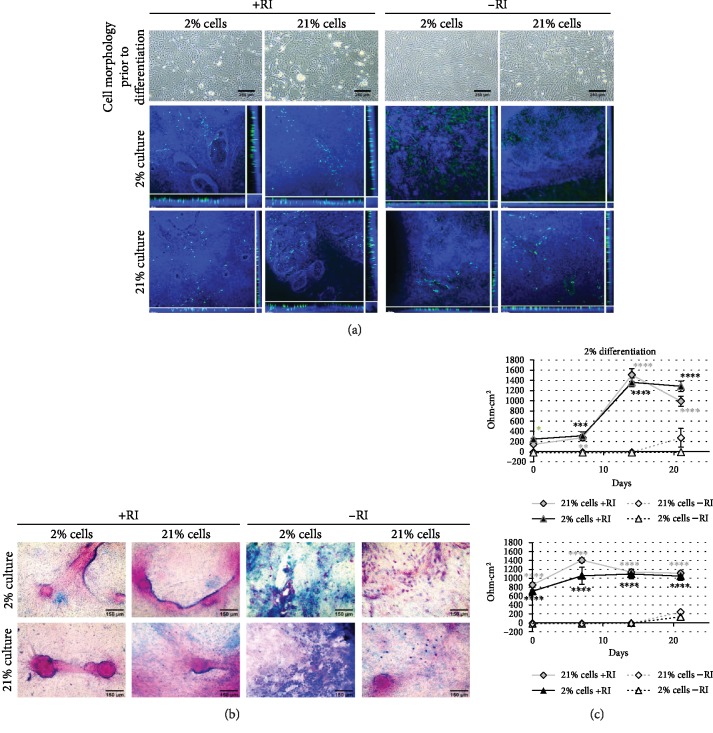
Early passage pAECs develop into differentiated monolayers with cilia and mucins at ALI, modulated by both RI supplementation and O_2_ level. (a) Upper: phase contrast micrographs of cells in submerged monolayer culture in the indicated conditions, prior to seeding for differentiation at ALI. Scale bar 250 *μ*m. Middle and lower: scanning laser confocal images of maximum intensity *x*-*y* and orthogonal *z* plane views of *β* IV tubulin (green) stained ALI cultures in the indicated culture conditions. DAPI nuclear counterstain (blue). Scale bar 100 *μ*m. (b) Alcian blue and PAS stained ALI cultures from the indicated conditions. Scale bar 150 *μ*m. (c) Transepithelial electrical resistance (*Ω*·cm^2^) of ALI cultures. Data are mean ± standard deviation, *n* = 3. ^∗^*p* ≤ 0.05, ^∗∗^*p* ≤ 0.01, ^∗∗∗^*p* ≤ 0.001, and ^∗∗∗∗^*p* ≤ 0.0001.

We next examined the differentiation capacity of our pAECs via an ALI model. To examine in more detail the effect of oxygen on differentiation versus culture, cells were differentiated in both the O_2_ condition they had been recovered in (2% or 21% cells) and in the alternative O_2_. The removal of mitogenic stimuli from ALI cultures has improved differentiation in rat cells [23]; therefore, we used a commercial medium, PneumaCult ALI (Stemcell Technologies), which induces excellent ALI differentiation in human cells [24]. We also differentiated in the presence and absence of RI to determine whether maintaining the cells in a proliferative state impacted subsequent differentiation. Firstly, the differentiation capacity of passage 2 cells was examined. At this early stage in culture, all conditions had cells of mixed morphologies (Figure 4(a)), upper), with RI-supplemented cells having clear epithelial colonies surrounded by fibroblastic cells in both O_2_ conditions, whilst cells cultured in the absence of RI were predominantly fibroblastic in appearance, again similar in both oxygen conditions. Cells were then cultured at ALI to induce differentiation. All cells in RI-supplemented media underwent ciliogenesis (Figure 4(a)), middle and lower left) with motile beating cilia visible by phase contrast microscopy in live cultures and distinct apical staining for *β* IV tubulin. This was also the case with –RI cells differentiated in 21% O_2_ irrespective of the previous isolation oxygen level (Figure 4(a), lower right). In contrast, cells recovered in either oxygen level but differentiated in 2% O_2_ in the absence of RI had more diffuse, cytoplasmic, *β* IV tubulin staining, with little evidence of apical projection. Differentiated monolayers were also stained with Alcian blue and PAS for acidic and neutral mucins, respectively (Figure 4(b)). All cells stained positively for acidic (light blue), neutral (pink), and mixed (purple/blue) mucins. RI-supplemented ALI cultures all showed the presence of airway-like structures, typically staining strongly with PAS and having a border of more acidic mucins. Cultures in the absence of RI had more obvious cellular staining for mucins indicating either greater mucous production or retention. Cultures originating with 2% O_2_ cells without RI displayed a tendency to produce acidic, rather than neutral, mucins indicated by the intense blue staining (not quantified).

For the duration of culture, the electrical resistance (*Ω*·cm^2^) was determined as a measure of monolayer integrity and the development of tight junctions (Figure 4(c)). In both 2% and 21% O_2_ cultures, there was a clear distinction between the TEER achieved in RI-supplemented cells compared to nonsupplemented cultures. Up to day 14, the TEER of unsupplemented cultures was effectively zero, increasing to a maximum of 273 *Ω*·cm^2^ in 21% O_2_ expanded cells differentiated in 2% O_2_ by day 21. In contrast, RI-supplemented cultures, with the single exception of day 0, 2% cultures of 21% cells, were always significantly higher. RI-supplemented 21% O_2_ cultures of both 2% and 21% expanded cells had much higher values at day 0 than the corresponding 2% cultures (710 ± 28 and 852 ± 55 *Ω* · cm^2^ in 2% cells and 21% expanded cells in 21% O_2_ differentiation compared to 247 ± 16 and 143 ± 30 *Ω* · cm^2^ in 2% cells and 21% expanded cells in 2% O_2_ differentiation); however, 2% cultures achieved similar high values of TEER by days 14 and 21 following an initial lag period. Notably, despite the low TEER values throughout the duration of culture, 21% differentiation of both 2% and 21% expanded cells without RI demonstrated clear evidence of mucociliary differentiation.

**Figure 5:**
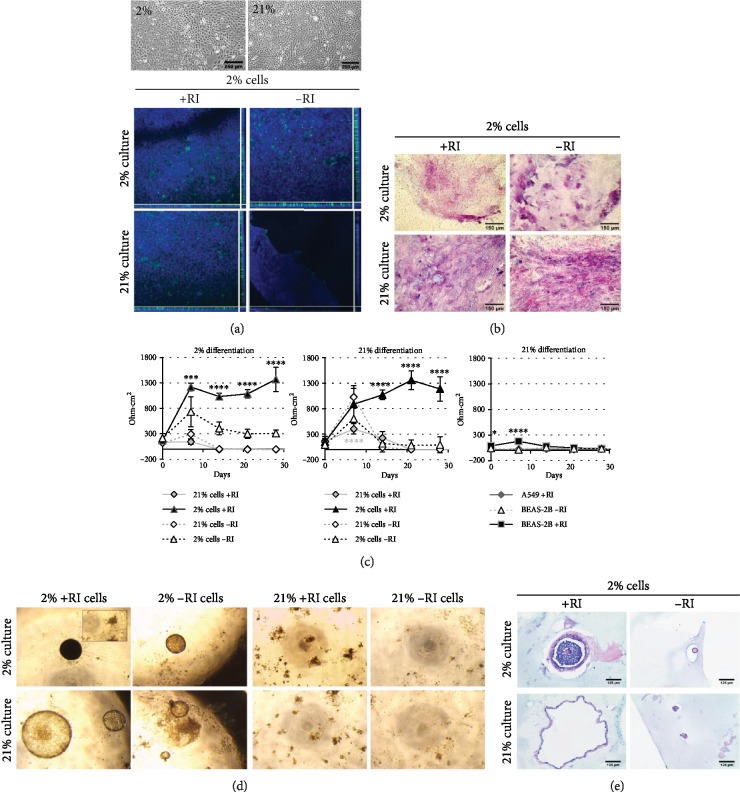
Low O_2_ culture-expanded cells and RI supplementation support the maintenance of monolayers at ALI in highly expanded pAEC cells. (a) Upper: phase contrast micrographs of cFAD+RI cultured cells in submerged monolayer culture, prior to seeding for differentiation at ALI. Scale bar 250 *μ*m. Middle and lower: scanning laser confocal images of maximum intensity *x*-*y* and orthogonal *z* plane views of *β* IV tubulin (green) stained ALI cultures in the indicated culture conditions. DAPI nuclear counterstain (blue). Scale bar 100 *μ*m. (b) Alcian blue and PAS stained ALI cultures from the indicated conditions. Scale bar 150 *μ*m. (c) Transepithelial electrical resistance (*Ω*·cm^2^) of ALI cultures. Data are mean ± standard deviation, *n* = 3. ^∗^*p* ≤ 0.05, ^∗∗∗^*p* ≤ 0.001, and ^∗∗∗∗^*p* ≤ 0.0001. (d) Images of Matrigel cultured organoids in the indicated conditions. (e) Alcian blue PAS stained formalin-fixed, paraffin-embedded sections (7 *μ*m) of the organoids shown in (d). Scale bar 125 *μ*m.

Extensively expanded cells (at passage 31), in the presence of RI to maintain an epithelial cell phenotype (where –RI refers to cells expanded in the presence, and differentiated in the absence, of RI), had their differentiation capacity explored. Previous comparisons of cell numbers and phenotype have demonstrated little difference between cells culture-expanded in 2% compared to 21% O_2_, and cells at the time of seeding appeared to be comparable in morphology in both O_2_ conditions (Figure 5(a), upper). Unlike the mixed populations seen with early passage, cultures no longer contained cells with a fibroblastic appearance and only very occasional squamous cells. Nevertheless, we saw striking differences when cells were placed at ALI. Cells expanded in standard 21% O_2_ conditions almost always failed to maintain a monolayer at ALI irrespective of the O_2_ level the differentiation was performed in and the presence or absence of RI. Intact monolayers were initially formed on inserts; however, cells then rounded up and detached, and the monolayer disintegrated. Other cultures were performed with a high degree of variability in our hands; in general, 2% O_2_ cells cultured in the absence of RI produced poor monolayers with regional gaps. Intact monolayers were consistently observed with 2% O_2_ expanded cells when the differentiation was carried out at 21% O_2_ and with continued supplementation with RI. ALI cultures showed development of apical *β* IV tubulin staining (Figure 5(a), middle and lower), and cilia could be seen under phase contrast. Cultures also stained positively for mucin production (Figure 5(b)), albeit at reduced levels compared to early passage cells. TEER values (Figure 5(c)) for these cultures reflected observations on the monolayer integrity with 21% expanded cells falling to, or below, starting TEER values by day 14 in line with disintegration of the monolayer. As with the general observations of the ALI cultures, high TEER values > 1000 *Ω* · cm^2^ were only maintained in 2% O_2_ expanded cells differentiated with the continued presence of RI. These values are comparable to those achieved with early passage cells in both 2% and 21% O_2_. Two cell lines commonly used for the generation of lung and airway models, A549 and BEAS-2B, were also cultured at ALI for 28 days, and the TEER was measured. A549 in the presence of RI failed to develop any significant TEER over the culture period. In contrast, BEAS-2B when cultured with RI had a significantly higher TEER at day 0 (89 *Ω*·cm^2^ compared to 42 *Ω*·cm^2^, *p* ≤ 0.05) increasing by day 7 (179 *Ω*·cm^2^ compared to 16 *Ω*·cm^2^, *p* ≤ 0.0001), after which the RI-supplemented culture value dropped to non-RI levels.

To establish whether the failure in monolayer integrity in 21% O_2_ expanded cells was a function of the ALI differentiation system or something integral to the cell biology, we differentiated our expanded cells as organoids in Matrigel. As with ALI cultures, all cells previously expanded in 21% O_2_ failed to sustain organoid integrity for the duration of the culture (Figure 5(d)) despite initially forming organoids indistinguishable from those with 2% expanded cells. 2% O_2_ expanded cells formed organoids in all conditions that were sustained for the duration of the culture; as with the most consistent performance at ALI in 21% with RI, organoids were also largest in those conditions. To confirm that the organoids were reminiscent of airway, they were fixed, paraffin embedded, sectioned, and stained with Alcian blue and PAS (Figure 5(e)); this confirmed that the organoids had hollow cores with thin walls. Specific mucin-positive cells were difficult to identify; however, organoids contained positively stained material (pink/dark blue/purple) indicating that mucins were being secreted into the organoid cavities.

**Figure 6:**
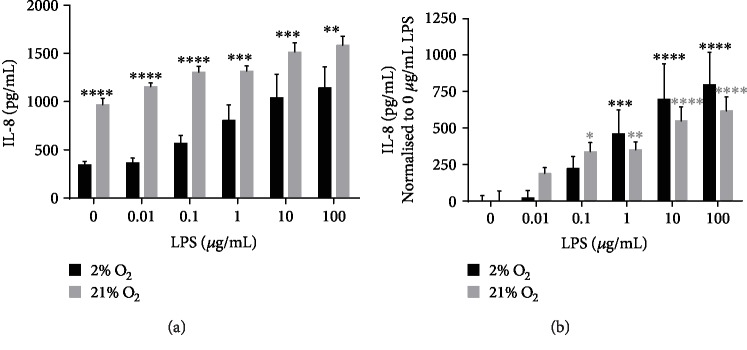
Expanded pAECs produce IL-8 in response to treatment with bacterial LPS. (a) IL-8 production (pg/mL) by pAECs during treatment of submerged cultures with bacterial LPS at 0, 0.01, 0.1, 1, 10, and 100 *μ*g/mL. (b) IL-8 produced by pAECs normalised to baseline (0 *μ*g/mL LPS treatment) levels. Data are mean ± standard deviation, *n* = 3. ^∗^*p* ≤ 0.05, ^∗∗^*p* ≤ 0.001, ^∗∗∗^*p* ≤ 0.001, and ^∗∗∗∗^*p* ≤ 0.0001.

In addition to its barrier function and mucociliary clearance, it is appreciated that the airway epithelium plays a key role in the immune response. We cultured our expanded cells in the presence of LPS to determine whether IL-8 was induced as a normal response. We saw that baseline levels of IL-8 in 21% O_2_ cultures were significantly higher than those in 2% cultured cells (966 ± 70 pg/mL in 21%, 343 ± 37 pg/mL 2%, *p* ≤ 0.0001) (Figure 6(a)) and remained significantly higher (0.01 and 0.1 *μ*g/mL LPS, *p* ≤ 0.0001; 1 and 10 *μ*g/mL LPS, *p* ≤ 0.001; 100 *μ*g/mL LPS, *p* ≤ 0.01) at all levels of LPS supplementation. In order to better distinguish the cell response to LPS, data were normalised to baseline levels in the absence of LPS (Figure 6(b)). 2% O_2_ cultured cells had significantly increased IL-8 secretion at levels of LPS treatment at or above 1 *μ*g/mL whilst 21% O_2_ cultured cells had significantly higher secretion at or above 0.1 *μ*g/mL LPS supplementation indicating a higher sensitivity to LPS in 21% O_2_ cultured cells. We noted that 2% O_2_ cultured cells showed a larger increase in IL-8 in response to higher LPS concentrations raising the possibility that 2% cultured cells are more responsive to a higher range of LPS concentrations perhaps due to lower baseline levels of secretion.

## 4. Discussion

Despite the need for a readily available cell source to bridge the gap between laboratory research and application, little work has been carried out to develop AEC culture with large, nonprimate mammals. Previous work over the last four decades, though limited, has focussed on dogs [25–27], sheep [28–33], horses [34], cows [35–39], and pigs [9, 27, 40–44] with little to no cell expansion being a common feature, as nonruminant omnivore pigs may prove to have the most similar biology to humans with comparable respiratory characteristics [45]. Here, we have successfully isolated and cultured mucociliary differentiation-competent pAECs that can reproducibly yield high, potentially unlimited, cells. Unlimited expansion is in feeder-free culture conditions, maintains an epithelial phenotype, and requires RI Y-27632 supplementation. Late passage pAECs retain naïve cell differentiation features including the capacity to polarise and maintain an ALI with sustained, high TEER values, undergo mucociliary differentiation, and to produce interleukin-8 in response to LPS. Interestingly, despite little apparent difference during culture expansion between 2% and 21% O_2_, cells subsequently displayed very different responses during differentiation culture. Cells expanded under standard 21% O_2_ conditions failed to maintain an ALI, and both ALI cultures and organoids disintegrated. Whilst the presence of RI was not essential during differentiation, we found that it was beneficial, improving the likelihood of the successful maintenance of ALI and the production of larger organoids.

Culture-associated phenotypic drift and the prospect of therapies reaching the clinic have been driving forces promoting a move away from the incorporation of undefined animal-derived products like FBS and bovine pituitary extract (BPE). Primary airway epithelial cells are frequently cultured in increasingly defined, serum-free formulations, for example, BEGM (Bronchial Epithelial Cell Growth Medium, Lonza), PneumaCult (Stemcell Technologies), LHC-9 (Gibco), and Airway Epithelial Growth Medium (PromoCell). Absolute “best-case” outcomes for culture in these defined media types suggest a maximum of 7-10 passages, with some associated decrease in the quality of differentiation subsequently in more aged cultures [24], although senescence as early as passage 2 may be seen [46]. We included two of these formulations (LHC-9 and Airway Epithelial Growth Medium) in our study. In agreement with other reports using defined media with human cells [18, 24, 47], pAECs typically underwent growth arrest within five passages. Furthermore, neither medium formulation supported the ongoing growth of cells when treated with RI although increases in recovered/starting cell numbers were seen. Cell populations in these media were initially very homogeneous and had excellent cobblestone morphology suggesting these media may be suitable for studies using only primary pAEC cultures where significant expansion is not necessary.

The growth of various human epithelial cell types in serum-containing medium leads to a halting of growth and onset of terminal differentiation, typically to a more squamous phenotype [22]. Specifically, the presence of transforming growth factor-*β* (TGF-*β*) in FBS has been implicated in this process [48, 49], particularly during low-density culture of cells [50]. Epithelial cells in culture, including those of the lung and airways, can also undergo an epithelial to mesenchymal transition (EMT) transdifferentiating and gaining characteristics of mesenchymal cells, such as a more fibroblastic appearance, expression of fibroblast markers, and fibronectin secretion; again, this can be mediated by FBS containing TGF-*β* amongst other EMT-inducing factors [51–53]. We observed examples of cells that are characteristic of both of these transitions in the FBS containing media cFAD, where mean cell size was significantly higher than in serum-free media and in agreement with previously reported values [22], likely as a result of a significant number of cells undergoing terminal squamous differentiation.

Culture of pAECs in cFAD medium without RI rapidly led to the loss of cell-cell contact and a population dominated by cells with an intermediate or more elongated, fibroblastic appearance (often apparent even at passage one), rather than the epithelial population was recovered at passage 0, or terminally differentiated squamous epithelial cells. Given the high epithelial cell dominance in the originally isolated populations, indicated by universal pan-cytokeratin expression, it is possible that these cells represent a population having undergone a rapid EMT, rather than the overgrowth of cultures with fibroblasts isolated from the airway. This rapid change in cell phenotype upon passage may be induced by the presence of EDTA during subculture that can activate noncanonical NOTCH1 signalling [52]. Interestingly, and in contrast to human cells in similar media formulations with and without feeder cells [18], we found that it is possible to expand these cells without RI with altered fibroblastic morphology, a considerable amount (beyond passage 30) with no signs of growth arrest; growth rates remain similar to RI-supplemented cultures throughout. Supplementing the cells with RI at a later time point had no recovery effect on the cell morphology.

Supplementation of cFAD with RI improved the numbers of cells obtained from the tissue during the initial recovery and expansion phase [53]. These cultures also underwent similar changes to those in cFAD without RI during early passages, with squamous and fibroblastic cells present, although to a lesser extent. Over subsequent passages, proliferative epithelial cells gradually became the dominant cell type in the cultures. Although the mechanism of action of RI in immortalisation has not been confirmed, Yugawa et al. show that prodifferentiation stimuli in epithelial cells, including the presence of EDTA at passage, may be initiating noncanonical NOTCH signalling via NOTCH1 and activated ROCK1 demonstrating a clear opportunity for ROCK inhibition [52]. Following the inclusion of RI in the cFAD media, we determined that a feeder layer was not necessary at any stage in order to either establish or maintain the cultures indefinitely. This has distinct benefits as the presence of contaminating feeder cells can interfere with the creation of ALI cultures where differential trypsinisation has not been 100% effective. This is in contrast to human breast and prostate CRCs where a feeder layer was absolutely required in order to initiate continuous cultures [18]. In agreement with the study defined, serum-free medium with RI was not able to support the establishment of continuous pAEC cultures [18, 24].

That we continued to see cells that had undergone both squamous and EMT-associated changes in RI-supplemented cultures does suggest that there may be some differences in our pAECs, either species related or as a result of the absence of feeder cells. The production of CRCs from human cells is described as a result of alterations across the whole population of cells rather than a selection of a subpopulation of cells [46]. Withdrawal of RI from previously supplemented cultures resulted in a slight reduction in proliferation rate, but in contrast to reports with human CRCs [54], no subsequent cessation of proliferation and, importantly, the concurrent maintenance of an epithelial phenotype indicating that the pAEC reprogramming was not conditional. These features have been recapitulated in cultures from multiple animal donors making it unlikely to be a result of spontaneous immortalisation as suggested elsewhere [54]. Inclusion of a feeder layer accompanies induction of telomerase in human CRCs which is insufficient to prevent senescence in the absence of Y-27632 [18]. Here, with pAECs, one or more components of the cFAD media appear able to induce sufficient telomerase activity to maintain indefinite proliferation, whilst RI may permanently prevent an irreversible EMT. Further investigation is required to confirm the mechanistic basis underpinning these observations.

pAECs at early passage underwent mucociliary differentiation at ALI which fell short of the ciliation levels found in native pig airways. Although the literature is very sparse, there are reports suggesting that cells cultured in conditions that induce robust differentiation in humans induce only poor differentiation in a number of animal species. In ovine [30], bovine [36], and porcine [41, 55] models, standard conditions resulted in thin, squamous monolayers with infrequent differentiated cells. In all three species, it was shown that supplementation of differentiation media with EGF improved ALI culture thickness resulting in a more robust pseudostratified layer of cells, and supplementation with retinoic acid significantly increased ciliation. These reports, although limited in number, suggest a clear route for the further optimisation of the culture conditions. In addition, differences were observed in mucous production, this being greater in the absence of RI. It is unclear at this stage whether this represents a more accurate model of the healthy epithelium or whether this is a result of the changes seen in cells in the absence of RI between passages 0 and 1 leading to mucous hyperplasia, a condition often correlating with other abnormalities, e.g., EMT in diseases such as asthma [56].

Y-27632 supplementation in early passage human cells reportedly has no effect on subsequent differentiation capacity, with no effect on numbers of ciliated or mucous-producing cells from either human or mouse cells [20, 54]. However, Gentzsch et al. report altered differentiation including reduced monolayer thickness, decreased ciliation, and altered electrical properties in both normal and cystic fibrosis human cells following application of the CRC protocol as early as passage 5 with a further reduction by passage 10 [47]. This is consistent with our observations where differentiation in highly expanded cells was at reduced levels in comparison to early passage cells. The loss of a conditional response to RI in the pAECs may play a role in this reduced differentiation capacity. The use of RI and feeders is described elsewhere as an inducer for P63 upregulation [18]. P63 is described as playing a role in negative regulation of differentiation, particularly tight junction and cilia formation [57]. The persistent upregulation of P63 may be maintaining cells in a basal stem cell state, thus inhibiting differentiation. Future studies will be required to determine if P63 inhibition promotes expanded pAEC differentiation.

Mammalian cell culture is typically performed in an ambient oxygen atmosphere in vitro which intuitively would be appropriate for airway cells and reflective of the environment experienced by surface tracheal epithelial cells in vivo (approximated as 19.5 kPa, assuming a negligible effect of airway surface liquid on diffusion where bronchiolar levels decrease to 11 kPa) [58]. It is not clear however what oxygen level is experienced by cells below the surface, i.e., for example, the basal cells. Peribronchial levels of O_2_ are 5-6 kPa and reliant on adequate perfusion from bronchial artery vasculature, not diffusion from airways [58]. Furthermore, a number of lung pathologies result in the presence of a more hypoxic environment in the lung, including cystic fibrosis, asthma, COPD, and areas subject to regional inflammation following injury; this can result in O_2_ levels as low as 0.3 kPa [59]. Long-term culture of cells involves substantial population expansion irrespective of somatic or stem characteristics. With a stem cell population of cells (be it a natural reservoir such as basal cells or CRCs with a stem cell nature), the potentially beneficial nature of reduced oxygen seen with many stem cell types in culture, possibly due to a more physiological recreation of their in vivo niche, can also be considered [60]. This, coupled with the negative consequences of transplantation of cultured cells to a hypoxic/hostile environment, means that it is desirable to understand cell behaviour in these potentially altered environmental conditions.

During the cell expansion phase, we saw no difference in phenotype between standard and reduced oxygen cultures. The differentiation of early passage cells cultured or differentiated in either oxygen level with RI was also similar, despite suggestions that the inhibition of differentiation in submerged cultures, particularly ciliogenesis, is as a result of the presence of media creating a hypoxic environment [61]. We did observe reduced ciliation in non-RI cells differentiated in reduced oxygen, potentially a result of the morphological change in the cells to a more mesenchymal type by this stage. Interestingly, switching these cells to a 21% O_2_ environment had a clear prodifferentiation effect with ciliation evident despite the low TEER of the monolayer. It is unclear at this stage whether this is a result of the promotion of growth and subsequent differentiation of a more epithelial subset of cells or whether the switch in O_2_ was reversing an EMT transition. We also saw increased mucous cell staining in these cells following culture and/or differentiation at low oxygen in accordance with previous reports that hypoxia causes mucous cell hyperplasia in human bronchial epithelial cell cultures [62]. It is therefore tempting to speculate that severely reduced oxygen association with a number of disorders of the airway is contributing to increased mucous production following mucous cell hypertrophy/hyperplasia, further contributing to the disease phenotype [63].

Oxygen level had a distinct effect on the behaviour of high-passage cells during differentiation. Air oxygen-expanded cells were generally unable to maintain a monolayer at ALI or to maintain organoids in Matrigel culture at either oxygen level. It is unclear why that is, although it may be that reduced oxygen cells are more tolerant of the switch from serum-containing growth media to serum-free, defined, differentiation media. The switch from serum-containing to serum-free media has been implicated in unreliable differentiation of a newborn pig tracheal cell line [42] and primary porcine epithelial cultures where detachment from the membrane was also observed with defined media cultures [9]. Nutrient availability has impacted bovine and ovine ALI cultures where low pore density inserts potentially restricted nutrient flux leading to reduced differentiation [29, 36]. Despite the interaction of oxygen with the cells constituting the airway being a key environmental feature, there is a limited body of work describing the explicit manipulation of the oxygen level though lowering the ambient oxygen level to 7-14% (bovine and ovine cells) is described as being of benefit to differentiation [29, 36].

In addition to mucociliary clearance, airway epithelial cells are intimately involved in the innate immune response via cytokine production including the neutrophil chemoattractant IL-8 [64]. We confirmed that as with human cell lines BEAS-2B and A549 [64, 65] and a newborn pig tracheal cell line [66], IL-8 was secreted when cells were challenged with bacterial endotoxin (LPS). Interestingly, we also noted a reduced baseline level of IL-8 secretion in 2% O_2_ cultures compared to 21% O_2_ cultures which may have implications for research in lung disorders with disrupted oxygen levels.

## 5. Conclusions

pAECs represent a readily available, cost-effective alternative to human or rodent AECs, which undergo mucociliary differentiation and form polarised monolayers capable of maintaining high TEER. The addition of the RI Y-27632 to cFAD medium supports unlimited feeder-free expansion of these cells, with the retention of differentiation capacity. Differentiation outcome, particularly of highly expanded cell populations, was altered depending on previous O_2_ culture expansion condition, O_2_ differentiation condition, and the ongoing presence or absence of RI. Further optimisation of culture conditions seems likely to improve differentiation outcome in both early and late passage pAECs with clear opportunities for the study of several key factors involved in lung disease, including EMT and the behaviour of cells in potentially severely hypoxic environments.

## Data Availability

All relevant data are within the paper.
